# Disseminated *Nocardia paucivorans* infection in a 64-year-old patient with first manifestation as an ulnocarpal forearm lesion

**DOI:** 10.1080/23320885.2026.2643076

**Published:** 2026-03-28

**Authors:** Peter Mohos, Lukas Mathys, Céline Bratschi, Georg Julian Claas, Philipp Honigmann, Marco Keller

**Affiliations:** ^a^ Department of Orthopaedic Surgery and Traumatology, Hand- and Peripheral Nerve Surgery, Kantonsspital Baselland, Bruderholz, Liestal, Laufen, Switzerland; ^b^ Handchirurgie de la Main, Biel/Bienne, Switzerland; ^c^ Institute of Infectiology, Kantonsspital Baselland, Bruderholz, Liestal, Laufen, Switzerland; ^d^ Department of Biomedical Engineering, Medical Additive Manufacturing Research Group (Swiss MAM), University of Basel, Allschwil, Switzerland; ^e^ Handzentrum Nordwestschweiz AG, Handzentrum Pratteln – Specialist Center for Hand and Peripheral Nerve Surgery, Pratteln, Switzerland; ^f^ Biomedical Engineering and Physics, Amsterdam UMC location University of Amsterdam, Amsterdam, The Netherlands; ^g^ Department of Orthopaedic Surgery and Traumatology, Hand- and Peripheral Nerve Surgery, Hospital Limmattal, Schlieren, Switzerland

**Keywords:** Central nervous system, forearm, immunocompromised, neurotropism, *Nocardia paucivorans*, skin

## Abstract

We report a 64-year-old immunocompromised patient receiving chemotherapy for metastatic colorectal adenocarcinoma who presented with a purulent forearm lesion. Surgical debridement identified *Nocardia paucivorans*. PET-CT and MRI revealed pulmonary and cerebral involvement. Escalated antimicrobial therapy achieved complete regression of brain lesions; the patient died 11 months later from underlying disease.

## Introduction

*Nocardia* spp. are filamentous, branching, Gram-positive, aerobic bacteria that are widely distributed in the environment, including soil, dust, stagnant water and decaying organic matter [1,2]. They act as facultative intracellular opportunistic pathogens and can cause both localized and disseminated infections, predominantly in immunocompromised hosts, although infections in immunocompetent individuals have also been described. In disseminated disease, the lungs, brain and skin are most commonly involved [3–6].

*Nocardia paucivorans*, first described in 2000, is a rare species associated with a broad clinical spectrum, ranging from localized cutaneous or pulmonary infections to disseminated disease with frequent central nervous system involvement [7,8]. Pulmonary infection following inhalation is considered the primary route of infection, with hematogenous dissemination occurring mainly in immunocompromised patients [4].

Because of its rarity and nonspecific clinical presentation, nocardiosis is frequently misdiagnosed and associated with substantial mortality in immunosuppressed patients. In addition, *Nocardia* species are slow-growing and difficult to culture, often resulting in delayed diagnosis, with colonies sometimes requiring two to four weeks to become detectable [8,9].

This case is unique in that a disseminated *Nocardia paucivorans* infection initially presented exclusively as an isolated cutaneous lesion of the forearm, preceding the detection of pulmonary and central nervous system involvement and occurring in the absence of systemic symptoms.

## Case report

**Figure 1.**
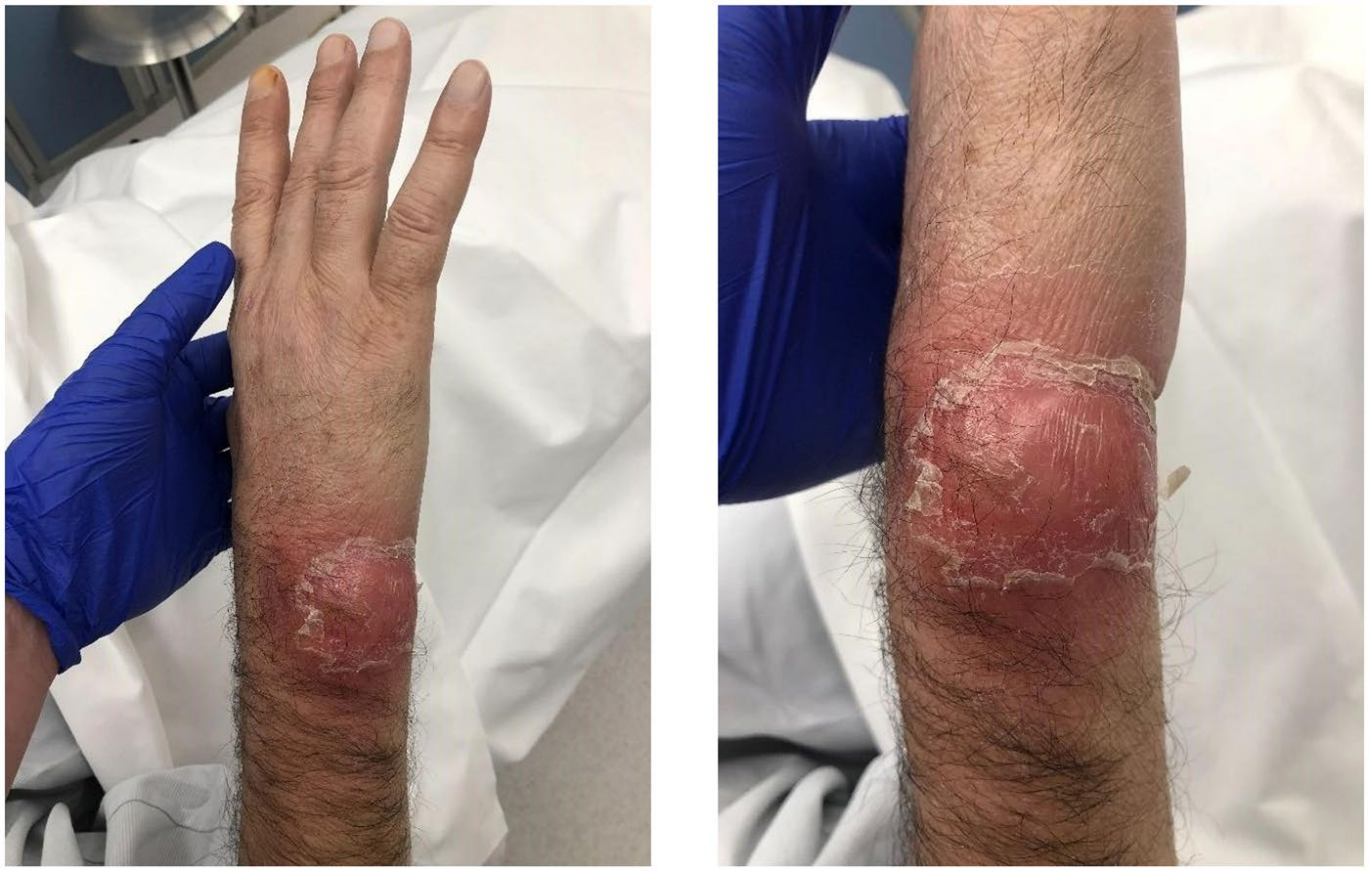
Erythema and fluctuating swelling on the ulnocarpal aspect of the right distal forearm at initial presentation, approximately four weeks after symptom onset.

A 64-year-old immunocompromised man presented to our emergency department with progressive erythema and fluctuating swelling on the ulnocarpal aspect of the right distal forearm (Figure 1). He reported that the lesion had first appeared approximately four weeks earlier, without any associated skin injury, and denied any previous trauma. He reported no other skin lesions, including irritation or pressure sores. He also denied fever or other systemic symptoms. The patient was undergoing chemotherapy for hepatic and pulmonary metastatic colorectal adenocarcinoma (FOLFOX and bevacizumab). His medical history included transanal endoscopic microsurgery and wedge resection of the right lower pulmonary lobe due to metastatic disease, as well as advanced liver fibrosis related to chronic alcohol abuse.

Upon admission, laboratory investigations revealed an elevated C-reactive protein level of 108 mg/L (reference < 1.0 mg/L), while the leukocyte count was within normal limits (6.2 G/L; reference 4.5–11.0 G/L). Plain radiographs of the forearm showed a soft tissue shadow in the ulnocarpal region without evidence of osteolysis or other bony pathology. Based on the clinical suspicion of a subcutaneous abscess, urgent surgical debridement was performed, and tissue samples were obtained for histological and microbiological analysis. The clinical timeline is summarised in Table 1.

**Table 1.** Clinical timeline of diagnosis, treatment and follow-up.

Postoperative timepoint	Clinical event
Day 0	Surgical debridement; biopsies obtained; empirical intravenous co-amoxicillin initiated.
Day 4	Discharge with oral co-amoxicillin.
Day 9	*Nocardia paucivorans* identified; switch to oral trimethoprim–sulfamethoxazole.
Day 10–15	Whole-body PET-CT and cranial MRI performed.
Day 15	Central nervous system involvement confirmed; intravenous ceftriaxone and amikacin initiated.
Month 2	Follow-up MRI demonstrating complete regression of the cerebral lesion.
Month 2 onward	Transition to long-term oral trimethoprim–sulfamethoxazole-based maintenance therapy.
Month 10	Last clinical follow-up with rehospitalisation due to hepatorenal syndrome and gastrointestinal bleeding.
Month 11	Death due to complications of the underlying disease.

**Figure 2.**
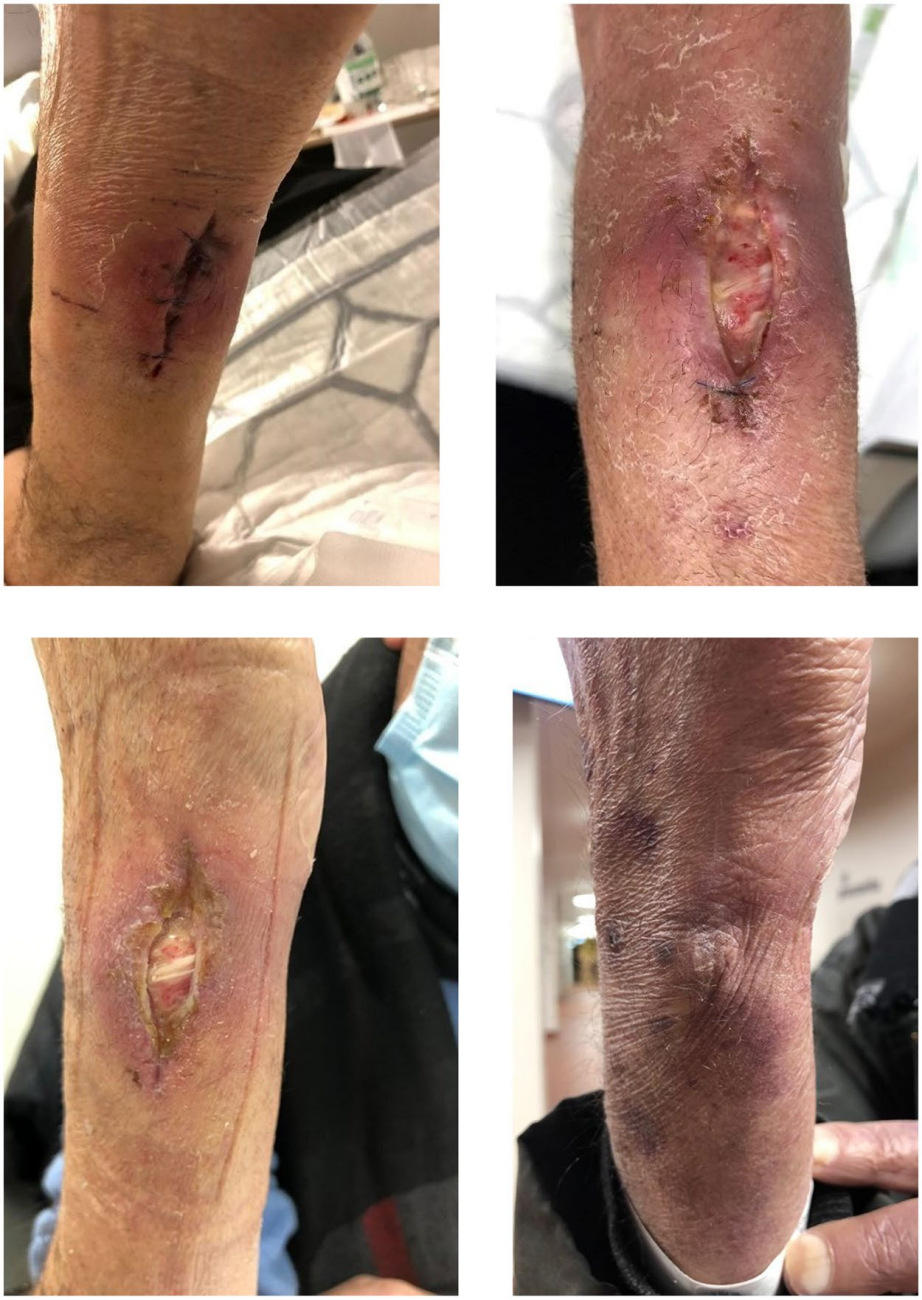
Postoperative wound healing progression following surgical debridement, demonstrating gradual wound healing under conservative management. *Upper left:* postoperative day 4; *upper right:* postoperative day 14; *lower left:* postoperative day 21; *lower right:* 10 months postoperatively.

Intraoperatively, an approximately 4-cm fenestrated, putrid supraretinacular mass consistent with a multiloculated subcutaneous abscess was identified. Empirical intravenous co-amoxicillin therapy was initiated immediately after surgery. After four days, the patient was discharged with regressing local symptoms and continued on oral co-amoxicillin. In the early postoperative period, partial suture removal was required due to increased local tension, resulting in wound dehiscence. Wound management was performed with regular dressing changes using absorbent foam dressings every two to three days in both outpatient and inpatient settings (Figure 2).

Microbiological cultures obtained intraoperatively identified pansensitive *Nocardia paucivorans* nine days after surgery (Table 2). Histopathological examination was consistent with infection. Following infectious disease consultation, antimicrobial therapy was switched from co-amoxicillin to oral trimethoprim–sulfamethoxazole.

**Table 2.** Antimicrobial susceptibility testing of *Nocardia paucivorans.*

Antimicrobial agent	MIC (mg/L)	Interpretation
Amoxicillin–clavulanic acid	0.75	S
Ceftriaxone	2.0	S
Imipenem	0.5	S
Clarithromycin	2.0	S
Doxycycline	0.19	S
Amikacin	0.5	S
Trimethoprim–sulfamethoxazole	0.016	S
Moxifloxacin	0.094	S
Linezolid	1.5	S
Minocycline	0.125	S

MIC: minimal inhibitory concentration; S: susceptible.

**Figure 3.**
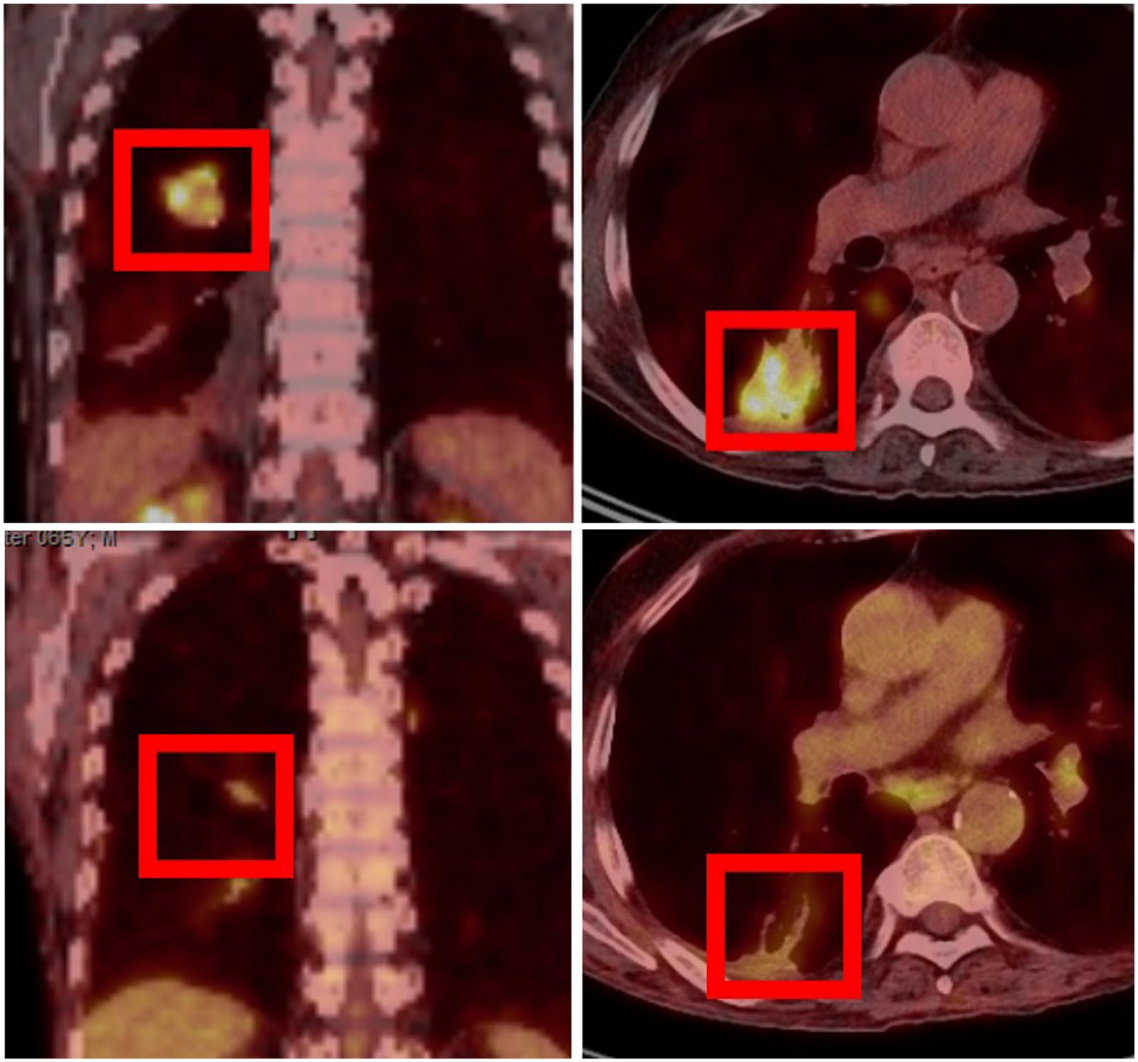
Pulmonary involvement on PET-CT before and after intravenous antimicrobial therapy. Whole-body PET-CT showing increased tracer uptake in the dorsobasal aspect of the right pulmonary lobe approximately two weeks postoperatively, prior to initiation of intravenous targeted antimicrobial therapy (upper row), and marked reduction in tracer uptake approximately two months after therapy initiation, consistent with treatment response (lower row).

**Figure 4.**
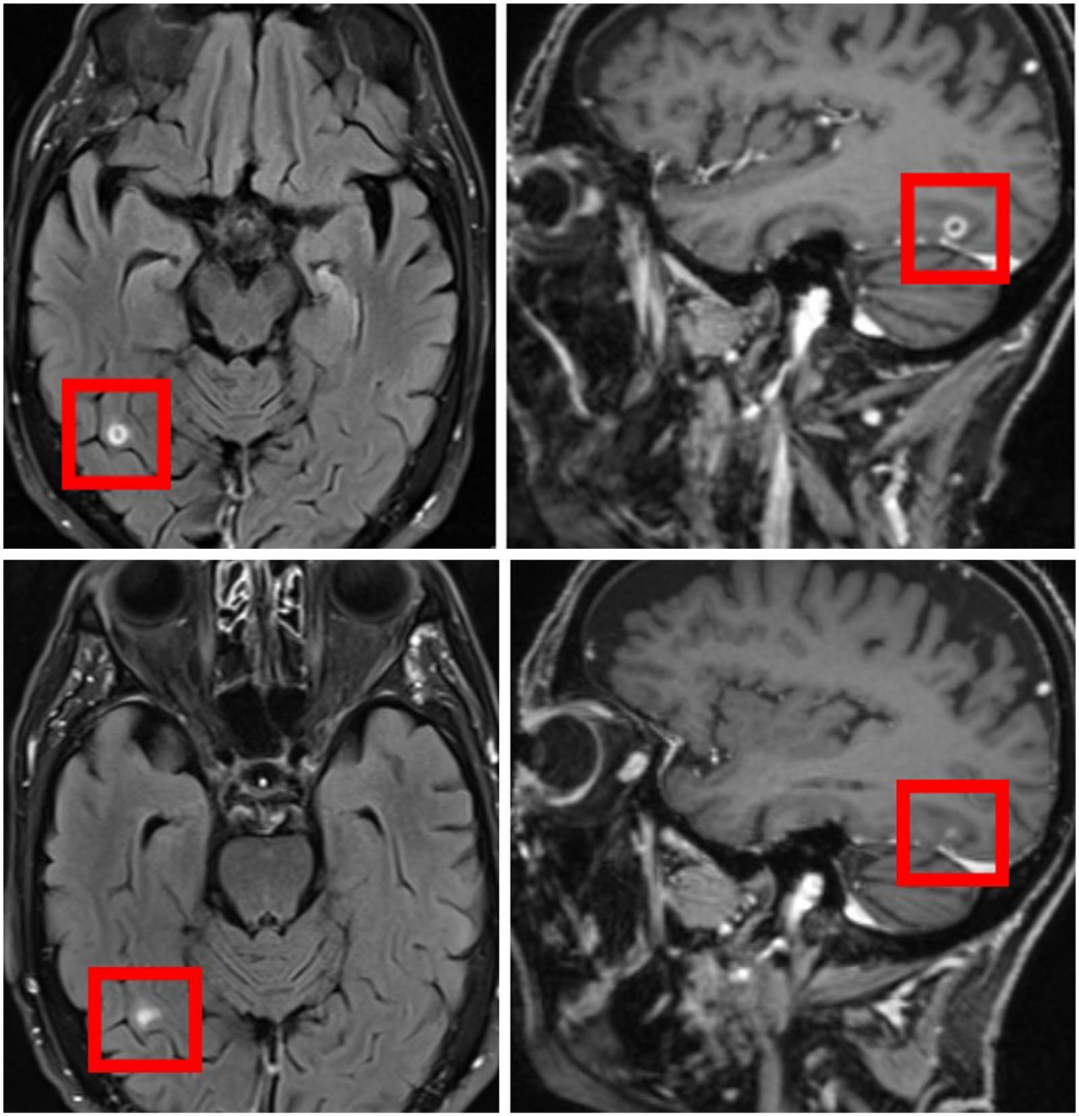
Cerebral involvement on cranial MRI before and after antimicrobial therapy. Cranial MRI demonstrating a solitary 0.6-cm ring-enhancing lesion in the right occipitotemporal gyrus approximately two weeks postoperatively, prior to initiation of intravenous antimicrobial therapy (upper row). Follow-up MRI approximately two months after therapy initiation shows complete regression of the cerebral abscess with residual gliosis (lower row).

Given the known neurotropism of *Nocardia* species and the patient’s immunocompromised status, complementary diagnostic imaging was recommended. Whole-body PET-CT and cranial MRI were subsequently performed approximately two weeks postoperatively. PET-CT demonstrated increased tracer uptake in the dorsobasal aspect of the right pulmonary lobe, suggestive of a presumptive pulmonary focus (Figure 3). Cranial MRI revealed a 0.6-cm ring-enhancing fronto-occipital lesion, radiologically consistent with a nocardial abscess rather than metastatic disease (Figure 4).

Based on the confirmation of central nervous system involvement, the patient was readmitted, and intravenous combination therapy with ceftriaxone (2 g once daily) and amikacin (15 mg/kg once daily) was initiated in accordance with infectious disease recommendations. Oral trimethoprim–sulfamethoxazole was discontinued during intravenous treatment. Intravenous antibiotic therapy was continued for six weeks.

Follow-up MRI after two months demonstrated complete regression of the cerebral abscess without new lesions (Figure 4). PET-CT showed a marked reduction in tracer uptake in the pulmonary lesion (Figure 3). Despite an initial delay in epithelialization, wound healing progressed satisfactorily under conservative management, and plastic surgical coverage was not required. Complete healing was achieved after approximately 12 weeks without contracture, preserving full range of motion of the wrist.

Following radiological remission of both cerebral and pulmonary lesions, antimicrobial therapy was transitioned to a long-term oral maintenance regimen in accordance with current recommendations for disseminated nocardiosis with central nervous system involvement [1]. During trimethoprim–sulfamethoxazole therapy, the patient developed a cutaneous hypersensitivity reaction, which was managed by temporary modification of the antimicrobial regimen. Subsequently, successful oral tolerance induction allowed continuation of trimethoprim–sulfamethoxazole-based therapy, with a planned total treatment duration of 12 months.

The final clinical follow-up occurred 10 months postoperatively, when the patient was rehospitalized due to hepatorenal syndrome and gastrointestinal bleeding. He passed away two weeks later from complications related to his underlying disease.

## Discussion

This case illustrates a disseminated *Nocardia paucivorans* infection in an immunocompromised patient, most likely originating from primary pulmonary inoculation with subsequent hematogenous dissemination to the central nervous system and the soft tissues of the forearm. At initial presentation, there were no clinical signs suggesting pulmonary or cerebral involvement, and no identifiable cutaneous entry point, which contributed to the diagnostic challenge.

Primary cutaneous inoculation with secondary dissemination must be considered in nocardiosis; however, available literature indicates that progression from intact skin to systemic disease is uncommon, particularly in the absence of trauma or a defined entry site [10]. Although immunosuppression increases the risk of dissemination [11], inhalation remains the predominant route of infection, with pulmonary involvement typically preceding secondary organ spread. In the present case, the absence of skin injury and the detection of a pulmonary focus strongly support inhalation as the most plausible primary route, with the cutaneous lesion representing a secondary manifestation of disseminated disease.

Among the various species within the genus *Nocardia*, *Nocardia brasiliensis* is the most commonly encountered pathogen, particularly in cutaneous and subcutaneous infections, and accounts for a substantial proportion of reported nocardiosis cases [12,13]. In contrast, *Nocardia paucivorans* is a rare species with only few reported clinical cases, associated with both localized and disseminated infections, frequently involving the central nervous system [14]. The largest available case series reported dissemination in approximately one-third of patients, predominantly affecting immunocompromised hosts, with frequent central nervous system involvement, supporting the hypothesis of a particular neurotropism of this species [14,15]. Our case aligns with these observations, despite the absence of neurological symptoms at presentation.

Host immune status plays a critical role in the pathogenesis of disseminated nocardiosis. Host defence against *Nocardia* relies primarily on cellular immunity, with T-lymphocytes being essential for long-term control of infection [16]. Gray et al. reported disseminated *Nocardia paucivorans* infection in a patient with hepatitis C, highlighting impaired T-cell function as a key predisposing factor despite apparent immunocompetence [14]. Similarly, *N. paucivorans* has been isolated from the cerebrospinal fluid of patients with T-cell deficiency [17]. Experimental and clinical data suggest that liver fibrosis induces a T-cell-suppressive immune environment, as described by Wu et al. [18]. In our patient, advanced liver fibrosis combined with systemic chemotherapy likely contributed to impaired cellular immunity, facilitating hematogenous dissemination.

Delayed diagnosis is common in nocardiosis due to its nonspecific clinical presentation [8,9]. In our case, the pathogen was identified within nine days of presentation, allowing timely initiation of targeted therapy. By contrast, a case series from Texas reported a mean diagnostic delay of three months (range, two weeks to 1.5 years) [19]. Similarly, a German case report described definitive identification only one month after biopsy using 16S rRNA sequencing [8].

*Nocardia* species are difficult to culture and require prolonged incubation when clinical suspicion exists, particularly in immunocompromised patients [12]. Brown-Elliott and Wallace highlighted polymerase chain reaction as a rapid diagnostic tool for detecting *Nocardia* species [10]. In addition, Mirsadraee et al. emphasized the value of imaging for identifying pulmonary involvement, supported by histopathological confirmation [20]. Given the risk of severe disease, empiric treatment with trimethoprim–sulfamethoxazole is commonly initiated in highly suspected cases [10].

Several pathogens may present with similar cutaneous and subcutaneous manifestations, necessitating consideration of differential diagnoses. Chronic lesions with swelling, induration, nodules, abscesses or draining sinuses may be observed in actinomycosis, botryomycosis, actinomycetoma and eumycetoma [12]. In addition, linear lymphangitis, a feature typical of nocardiosis, can also be seen in infections caused by *Sporothrix*, *Mycobacterium marinum*, *M. kansasii*, *Leishmania*, *Coccidioides*, *Cryptococcus*, lupus vulgaris and tularemia [12].

In the present case, microbiological identification from intraoperative biopsies prompted adjustment of antimicrobial therapy, and oral trimethoprim–sulfamethoxazole was initiated. Given the patient’s immunocompromised status and the known neurotropism of *Nocardia* species, complementary imaging led to the diagnosis of disseminated nocardiosis with central nervous system involvement.

Targeted antimicrobial therapy is essential in disseminated nocardiosis [8]. Although *Nocardia* species are generally susceptible to trimethoprim–sulfamethoxazole, resistant *Nocardia paucivorans* strains have been reported [21]; in our case, the isolate was pansensitive (Table 2). According to current recommendations, disseminated nocardiosis with central nervous system involvement requires combination intravenous therapy for four to six weeks, followed by prolonged oral maintenance therapy [1]. In the present case, guideline-based escalation to intravenous ceftriaxone and amikacin resulted in complete radiological regression of cerebral lesions, underscoring the importance of early recognition and timely therapeutic adjustment in immunocompromised patients.

## Conclusions

When patients present with atypical, atraumatic cutaneous lesions, nocardiosis should be considered as a potential diagnosis, particularly in high-risk groups such as immunocompromised individuals or patients with T-cell dysfunction. Given the neurotropism of *Nocardia* species and the high mortality associated with disseminated disease, complementary imaging – especially of the central nervous system – should be performed once microbiological confirmation is obtained, even in the absence of neurological symptoms.
